# Myocardial extracellular volume expansion in patients with hypertension

**DOI:** 10.1186/1532-429X-15-S1-O110

**Published:** 2013-01-30

**Authors:** Tomas G Neilan, Francois-Pierre Mongeon, Otavio R Coelho-Filho, Ravi Shah, Ciaran J McMullan, Siddique Abbasi, Eri Watanabe, Bobby Heydari, Ron Blankstein, Raymond Y Kwong, Michael Jerosch-Herold

**Affiliations:** 1 Cardiology, Brigham and Women's Hospital/Massachusetts General Hospital, Boston, MA, USA; 2 Cardiology, Massachusetts General Hospital, Boston, MA, USA; 3 Cardiology, Tokyo Women's Medical University, Tokyo, Japan; 4 Cardiology, Montreal Heart Institute, Montreal, QC, Canada; 5 Internal Medicine, State University of Campinas (UNICAMP), Sao Paulo, Brazil; 6 Nephrology, Brigham and Women's Hospital, Boston, MA, USA

## Background

Hypertension is associated with the development of myocardial fibrosis with a subsequent increase in the myocardial extracellular volume (ECV). T1 measurements pre and post a contrast cardiac magnetic resonance (CMR) study provide a robust non-invasive method for quantification of the ECV. We aimed to determine whether the CMR-derived ECV can differentiate healthy volunteers from patients with systemic arterial hypertension.

## Methods

We performed a CMR study including T1 measurements in 145 patients with hypertension. CMR variables were compared to a group of age-, gender-matched healthy volunteers. Patients with hypertension were followed for the development of adverse cardiovascular outcomes (death, heart failure admission, and atrial fibrillation). Values were compared to age- and gender-matched controls.

## Results

Study patients were 66% male with a median age of 58 years (range 36-85), had a BMI of 30±4 kg/m2, a left ventricular (LV) end-diastolic volume of 155±35 ml, an LV mass index of 68±20 gms/m^2^, and a LV ejection fraction of 61±6%. In comparison to healthy controls, patients with hypertension had elevated LV volumes, LV mass, and left atrial volumes. The ECV was elevated in patients with hypertension as compared to healthy controls (hypertension vs. healthy controls, 0.34±0.03 vs. 0.29±0.03, p<0.001). In patients with hypertension, there was a positive association between the ECV and indexed left atrial volume (r=0.46, p<0.01), and the LV mass index (r=0.45, p<0.01) and a negative association between the ECV and early mitral annular relaxation (E', r=-0.55, p<0.001). The ECV was similar in males and females with AH (0.34±0.03 vs. 0.33±0.03, p=0.18). During a median follow-up time of 18 months, there were 52 events. In univariable analysis, the ECV (hazard ratio (HR) 1.35, 95% CI 1.21-1.51, chi-squared 24.6, p<0.0001), indexed maximal LA volume (HR 1.02, 95% CI 1.00-1.03, chi-squared 4.71, p=0.03), and the LV mass index (HR 1.02, 95% CI 1.00-1.03, chi-squared 5.25, p=0.02) provided the strongest association with the adverse clinical events. In a multivariate model, the ECV provided the strongest adjusted association with the composite endpoint. Each 10% increase in the ECV was associated with a 24% increased risk of adverse events.

## Conclusions

In comparison to healthy volunteers, the ECV is increased in patients with hypertension, and is associated with structural and functional abnormalities. In follow-up, the ECV provided the strongest adjusted association with a composite end-point of death, heart failure hospitalization and late AF recurrence.

## Funding

Dr. Neilan is supported by an American Heart Association Fellow to Faculty grant (12FTF12060588) and previously by an NIH T32 Training Grant (T32HL09430101A1). Dr. Mongeon receives financial support for research from the Montreal Heart Institute Foundation, Montreal, Canada. Dr. Kwong receives salary support from a research grant from the National Institutes of Health (R01HL091157).

Dr. Jerosch-Herold is supported in part by a research grant from the National Institutes of Health (R01HL090634-01A1).

**Figure 1:**
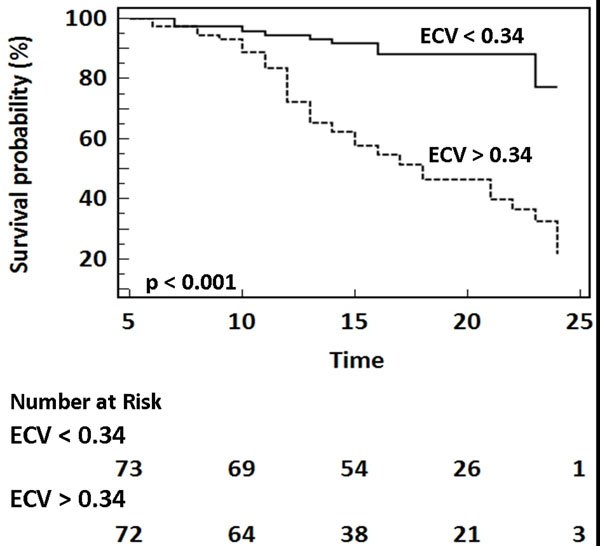
Kaplan-Meier curve showing the event-free survival in patients separated according to the median ECV value of 0.34. Results were compared using a Log-Rank test. Events were a composite of death, heart failure admission, or late recurrence of atrial fibrillation. Time is recorded in months.

